# Cyanide Hydratase Modification Using Computational Design and Docking Analysis for Improved Binding Affinity in Cyanide Detoxification

**DOI:** 10.3390/molecules26061799

**Published:** 2021-03-23

**Authors:** Narges Malmir, Najaf Allahyari Fard, Yamkela Mgwatyu, Lukhanyo Mekuto

**Affiliations:** 1 National Institute of Genetic Engineering and Biotechnology (NIGEB), Shahrak-e Pajoohesh km 15, Tehran-Karaj Highway, Tehran 14965/161, Iran; nargesmalmir1@gmail.com (N.M.); allahyar@nigeb.ac.ir (N.A.F.); 2 Department of Chemical Engineering, University of Johannesburg, Doornfontein, Johannesburg 2094, South Africa; yamkelamgwatyu@gmail.com

**Keywords:** *Trichoderma harzianum*, cyanide, cyanide hydratase (CHT), mutation, protein engineering, docking analysis

## Abstract

Cyanide is a hazardous and detrimental chemical that causes the inactivation of the respiration system through the inactivation of cytochrome c oxidase. Because of the limitation in the number of cyanide-degrading enzymes, there is a great demand to design and introduce new enzymes with better functionality. This study developed an integrated method of protein-homology-modelling and ligand-docking protein-design approaches that reconstructs a better active site from cyanide hydratase (CHT) structure. Designing a mutant CHT (mCHT) can improve the CHT performance. A computational design procedure that focuses on mutation for constructing a new model of cyanide hydratase with better activity was used. In fact, this study predicted the three-dimensional (3D) structure of CHT for subsequent analysis. Inducing mutation on CHT of *Trichoderma harzianum* was performed and molecular docking was used to compare protein interaction with cyanide as a ligand in both CHT and mCHT. By combining multiple designed mutations, a significant improvement in docking for CHT was obtained. The results demonstrate computational capabilities for enhancing and accelerating enzyme activity. The result of sequence alignment and homology modeling show that catalytic triad (Cys-Glu-Lys) was conserved in CHT of *Trichoderma harzianum*. By inducing mutation in CHT structure, MolDock score enhanced from −18.1752 to −23.8575, thus the nucleophilic attack can occur rapidly by adding Cys in the catalytic cavity and the total charge of protein in pH 6.5 is increased from −6.0004 to −5.0004. Also, molecular dynamic simulation shows a stable protein-ligand complex model. These changes would help in the cyanide degradation process by mCHT.

## 1. Introduction

Cyanide is extremely toxic to most living organisms and nowadays, the contamination of the environment with hazardous and toxic chemicals like cyanides is one of the most important problems facing the industrialized world. Cyanide can strongly bind to metalloproteins like cytochrome c oxidase, which is responsible for oxidative phosphorylation [1,2]. Because of the chemical properties of the cyano group (-C≡N), cyanide can be observed in various forms in the environment. Depending on the pH, free cyanide can be found in the form of CN^−^ ion in solution or cyanhydric acid gas, while nitriles (R-C≡N) result from the reaction of free cyanide and organic compounds [3]. Metals such as copper, zinc, nickel, cadmium, or iron can react and form metal-cyanide complexes, and these complexes are usually very stable and venomous [4]. Anthropogenic Industrial activities such as metal processing, gold mining, steel, and aluminum manufacturing, electroplating, jewelry producing, nitrile pesticides used in agriculture, and radiographic film recovery are the main sources of cyanide contamination in the environment, with an estimated 18 billion liters of cyanide-containing effluents produced yearly. Plants such as cassava, almonds, and peaches contain cyanide glycosides in their seeds, fruits, leaves, and roots [1,5]. However, the contribution of cyanide pollution from these natural resources to the environment is minimal compared to the anthropogenic activities such as mining and extraction metallurgy practices. Cyanide is currently removed using three main methods: biological, chemical, and physical methods. Among these methods, biological remediation has been known to be the most robust, cost-effective, and environmentally benign method for cyanide treatment. Microorganisms such as bacteria and fungi can treat cyanide compounds by producing cyanide-degrading enzymes. For example, bacterial species such as *Pseudomonas fluorescens, Bacillus pumilus,* and *Pseudomonas putida,* as well as fungal organisms such as *Fusarium solani* and *Trichoderma spp*., have been well-studied in cyanide biodegradation [6,7,8]. One of the biocontrol agents that is used against plant fungal diseases is *Trichoderma spp*., an organism that can degrade cyanide compounds by using rhodanese and cyanide hydratase, which are two of the several cyanide-degrading enzymes [9].

The enzymes utilize a variety of pathways to decontaminate cyanide species and these include: (1) hydrolytic, (2) oxidative, (3) reductive, (4) substitution/transfer, and (5) synthesis pathway. *Trichoderma* spp. utilizes both rhodanese and cyanide hydratase, which uses both the hydrolytic and substitution/transfer pathway depending on the physicochemical conditions. However, *Trichoderma harzianum* utilizes the hydrolytic pathway for the degradation of cyanide. In this pathway, cyanide is degraded to form ammonium and formic acid.

**Figure 1:**
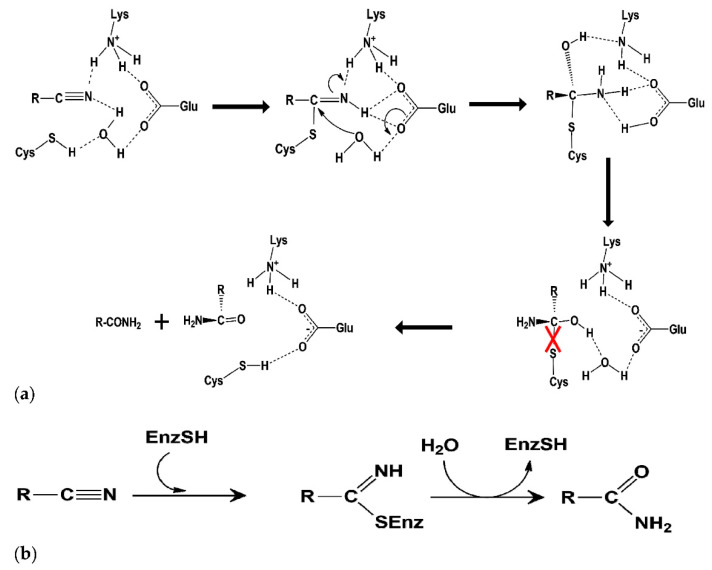
(**a**) Putative catalytic mechanism of cyanide hydratase, and (**b**) reaction catalyzed cyanide degradation.

One of the most important emerging tools that use genomic, transcriptomic, and proteomic techniques to study biodegradation of cyanurated wastes is Cyan-omics. This tool uses genome analysis of cyanide-degrading microorganisms to provide information on the metabolic maps of organisms and makes it possible to investigate bioremediation in industrial wastewaters. Thus, using this tool for studying and identifying cyanotrophic microorganisms and comparing genes that are involved in cyanide detoxification demonstrates a new way to extend our knowledge on cyanide biodegradation pathways [10]. Recently, metagenomics was applied for the genomic analysis of a cyanide-degrading population of uncultured microorganisms [10,11]. Due to the increasing demand for cyanide-rich waste degradation, the genetic modification of *Trichoderma spp.* for an increased rhodanese and cyanide hydratase activity has been performed for cyanide bioremediation [6]. The amino acid sequence of cyanide dihydratase in *Flavobacterium indicum* MTCC6936 has 50% and 43% sequence identity with the putative amino acid sequence of *F. indicum* and cyanide dihydratase isolated from *Bacillus pumilus* respectively, and its catalytic triad was Glu at 46, Lys at 130, and Cys at 164th position [12]. Cyanide hydratase is one of the nitrilase superfamily proteins and the structure of these proteins have an active site with a triad of residues, Glu-Lys-Cys, which is essential for their function and enhances the performance of proteins [13]. The mutation of any residue of this triad will result in loss of function in this protein family [14]. The reaction of the nitrile and cyanide degradation in the nitrilase superfamily takes place via the formation of a tetrahedral intermediate in the active site. The tetrahedral intermediate can be hydrolyzed using the active site of cyanide hydratase and its pathway eliminates the formal thiol of Cys to produce an amide, and Glu is likely to be the proton provider (Figure 1) [15]. All of the cyanide degradation reactions are initiated by a nucleophilic attack using the active site cysteine residue on the cyano or carbonyl carbon atom. The glutamate in the catalytic triad is likely to play the role of a catalytic base, while the lysine provides electrostatic stabilization as a part of the nitrilase equivalent of the oxyanion hole [16].

Some works are focused on protein engineering of a cyanide-degrading enzyme to expand the reaction range to afford more additional pathways for nitrile and cyanide degradation [15]. Also, this study reconstructed the active site of cyanide hydratase isolated from *Trichoderma harzianum* by computational design for increased usage in cyanide degradation in different industrial wastewaters. The new construction of cyanide hydratase was compared with the wild-type strain and the ligand docking was analyzed in both enzymes.

## 2. Results

### 2.1. Multiple Sequence Alignment and Sequence Analysis

**Figure 2:**
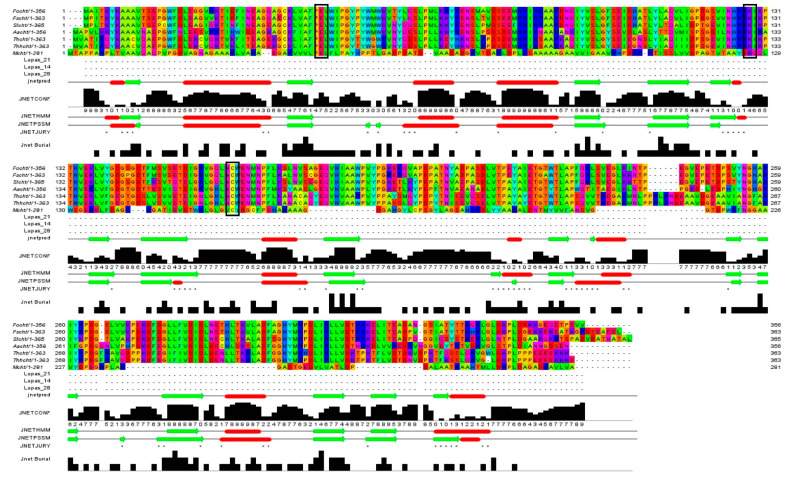
Conserved residues and the catalytic triad (black boxes) of cyanide hydratase isolated from fungal species.

Cyanide hydratase (CHT) contained 363 residues and its molecular weight was 41.2 KDa. The calculated isoelectric point (PI) of CHT was 5.77, which indicates the acidic nature of the protein. The aliphatic index, which is demonstrated as volume occupied by residues such as alanine, valine, leucine, and isoleucine, defines thermal stability of globular proteins [17]. The high value of aliphatic index in CHT (83.00) suggests probable stability of proteins in a wide range of temperatures. The instability index describes a factor that estimates the stability of protein in a test tube. A value above 40 shows the probable stability of protein in a test tube and vice versa [18]. In this study, this value for CHT protein was calculated to be 39.62, indicating the probable stability of CHT in a test tube. The Grand average of hydropathicity index (GRAVY) were estimated to be −0.435. A negative value indicates high affinity of the molecule to water. Functional domain analysis using Pfam, InterPro, and Conserved Domains Database (CDD) revealed this protein as an amidase that belong to the Nitrilase superfamily. The cyanide hydratase protein of indigenous *Trichoderma harzianum* was aligned with this protein of six other fungal species, and their identity in the catalytic triad has been observed (Figure 2). Three residues in the active site of cyanide hydratase including Glutamic acid, Lysine, and Cysteine are conserved, and other conserved residues are indicated in Figure 2. Conserved residues of the active site were E48, K130, and C165 in cyanide hydratase of indigenous *Trichoderma harzianum*.

**Figure 3:**
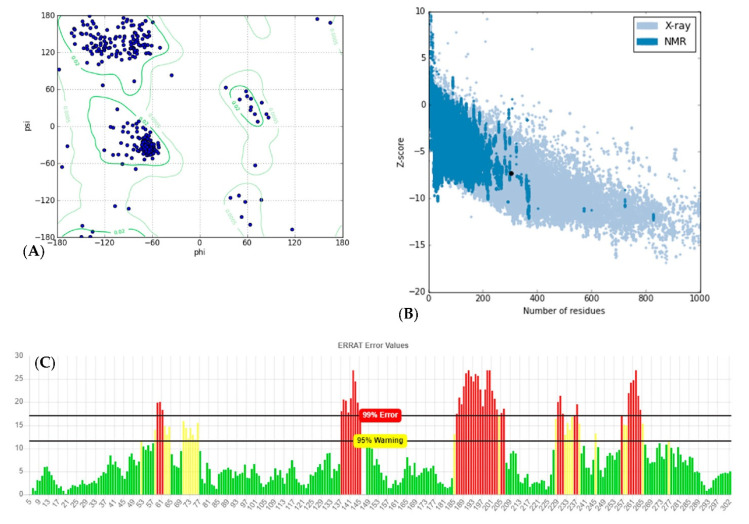
Structural validations of predicted CHT model: (**A**) Ramachandran plot: as shown in plot, 98.9% of residues are in the most favored and allowed regions, while 1.1% are within the disallowed region (3 residues). (**B**) Z-plot: Z-score of the model is shown as a black dot within the plot region, which is in the range of the native model with the same size. (**C**) ERRAT evaluation plot showing the quality of model based on non-bond interactions. ERRAT quality score was calculated to be 76.610.

Analyzing for Verify3D score [19], 63.4 percent of amino acids produced a score of above 0.2 and 14 amino acids had a negative score, indicating a good sequence-to-structure agreement. In addition, Ramachandran plot analysis by PROCHECK server [20] showed that 98.9 percent of residues were within the most favored and allowed regions, while 1.1 percent were within the disallowed region (Figure 3A). This reconfirms that backbone dihedral angels occupied appropriate position in the predicted model. Finally, the energy of the structure was evaluated using the ProSA web server [21]. This analysis showed that the z-score of the model is within the range of scores typically found for native proteins of similar size (Figure 3B). ERRAT is a so-called “overall quality factor” and this quality score that takes non-bonded atomic interactions into consideration was 76.610, which is greater than 50, set as a threshold for high-quality structures [22] (Figure 3C).

### 2.2. Homology Modeling and Docking Analysis

**Figure 4:**
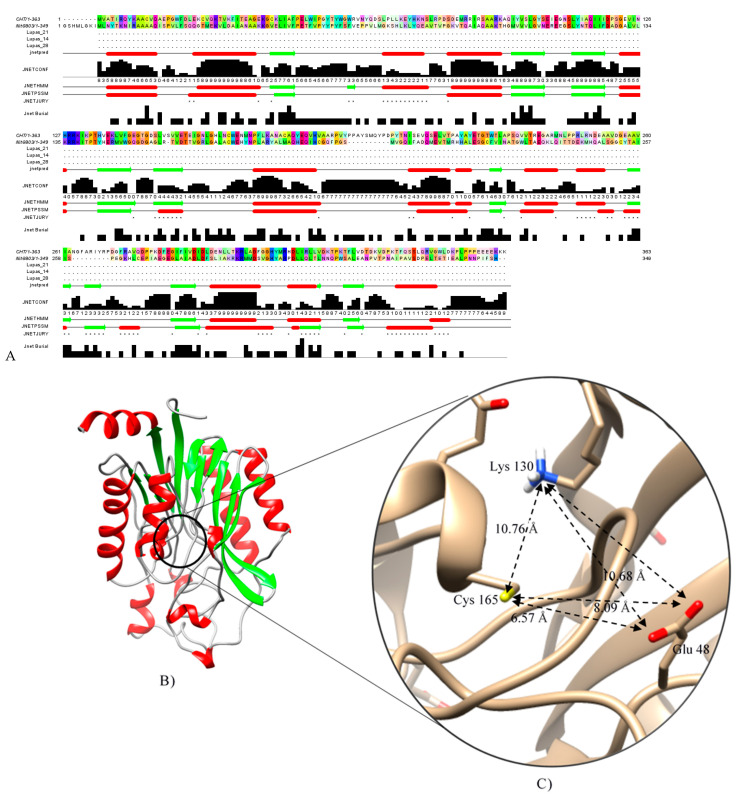
(**A**) CHT and Nit6803 and alignment of their secondary structural elements. (**B**) Tertiary structure of cyanide hydratase model, and (**C**) its catalytic triad predicted by Chimera software [24].

**Figure 5:**
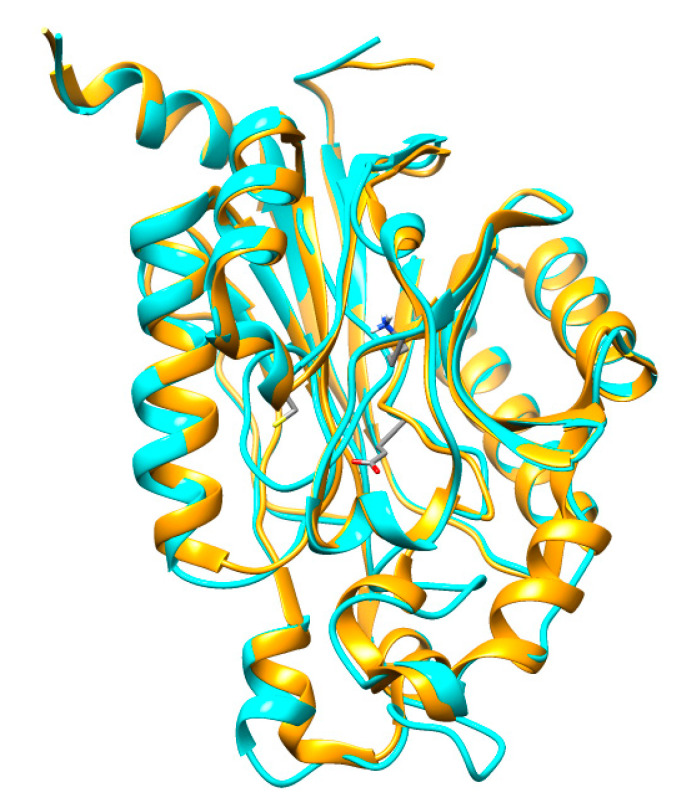
Superimposed structures of CHT (blue) and Nit6803 (golden) three-dimensional (3D) structures.

A BLAST search was conducted with CHT amino-acid sequence to find a known crystal structure of *Syechocystis sp.* Nitrilase (Nit6803) (PDB Acc. No.: 3WUY chain A) determined by X-ray diffraction at 3.1 Å resolution [23], which was used as a template for homology modeling. The protein sequence of CHT showed 29.07% amino acid identities in a coverage of 91% to Nit6803 (Figure 4A), and the secondary structural elements were conserved in both sequences (Figure 4A). Consequently, the tertiary structure of CHT of *Trichoderma harzianum* was generated using the SWISS-MODEL workspace, as shown in Figure 4B. The quality of the predicted model was analyzed with several evaluation methods. The superimposition of the modeled protein structure with the template showed the root-mean-square deviation (RMSD) value of 0.06 nm (Figure 5). Tertiary structure of cyanide hydratase from *Trichoderma harzianum* was drawn using the molecular modeling system UCSF Chimera (Figure 4B) and its catalytic triad (catalytic cavity) was conserved (Figure 4C). This protein belongs to the nitrilase superfamily, and the predicted structure includes 10 strands of parallel β-sheets that are surrounded by 11 a-helices (Figure 4B). These actions were performed for other proteins and their model was built.

**Figure 6:**
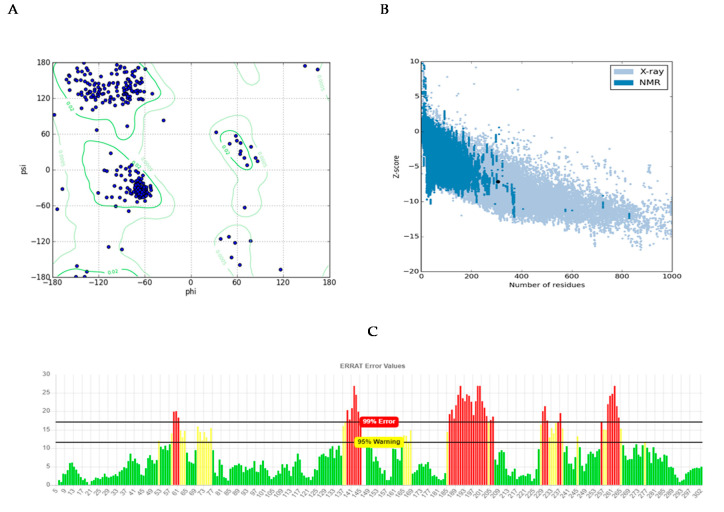
Structural validations of predicted mCHT model: (**A**) Ramachandran plot: as shown in the plot, 98.9% of residues are in the most favored and allowed regions, while 1.1% are within the disallowed region (3 residues). (**B**) Z-plot: Z-score of the model is shown as a black dot within the plot region, which is in the range of native model with the same size. (**C**) ERRAT evaluation plot showing the quality of model based on non-bond interactions. ERRAT quality score was calculated to be 75.2542.

After predicting models for cyanide hydratase, molecular docking simulation by the Molegro Virtual Docker (MVD) version 4.0.2 [25] was used to analyze and compare protein docking in CHT, its mutant, and other models of cyanide hydratase in different fungi (Figure 6). To do this, a cavity encompassing E48, K130, and C165 in the predicted model was assumed as the CN^-^ binding site and the ligand dataset was docked to the CHT. Evaluation of the outcomes indicated that cyanide hydratase of *Fusarium oxysporum* has the highest MolDock score among other proteins (Table 1). The score mimics the potential energy change, when the protein and ligand come together. This means that a very negative score corresponds to a strong binding and a less negative or even positive score corresponds to a weak or non-existing binding. Based on these predictions, the residues of CHT that have a close relationship with the catalytic cavity in terms of position and reaction can be determined by Molegro Virtual Docker software and analyzed for designing a mutant (Table 2). As it was described in Figure 1, the distance between the nitrogen atom of cyanide and the hydrogen atom of Lys (A), carbon atom of cyanide and sulfur atom of Cys (B), and oxygen atom of Glu and hydrogen atom of Cys (C) in the active site are critical at the first stage of cyanide degradation by cyanide hydratase. Formation of certain orientation and interaction for nucleophilic attack and amidase activity of the enzyme in the nitrilase superfamily are necessary. In addition to the MolDock score, these distances in the catalytic triad should be optimal and they were measured (Table 3). It was revealed that the cyanide hydratase of *Fusarium oxysporum* and *Trichoderma harzianum* that have the highest MolDock score show the optimal distance of (A) and (B), and these distances are critical for their action. Some reports reveal several residues that were changed successfully. In a study, two mutations of F168V and L201N were combined to produce a nitrilase mutant that demonstrated specific activity 15.3-fold greater than the native enzyme. Residue 168 is within the catalytic domain of the nitrilase and is separated from the catalytic cysteine by only three residues, it is not surprising that changing this bulky phenylalanine would have an impact on activity [26]. Several reports show that the inactivation of the amidase domain by mutation of the catalytic Cys, Lys, and Glu can inactivate cyanide hydratase [27].

### 2.3. Designing a Mutant of Cyanide Hydratase, Modeling, and Comparing with the Native Enzyme

**Figure 7:**
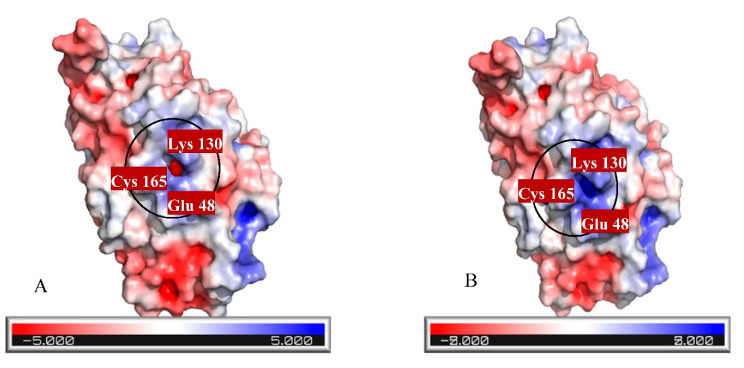
Electrostatic potential molecular surface of (**A**) wild-type and (**B**) mutant proteins.

Three strategies were used for constructing a mutant protein in our study: (1) increasing nucleophilic attack by adding Cys in the catalytic cavity, (2) increasing positive charge in the active site, and (3) eliminating bulky residues for creating a more spacious cavity to substrate entrance. As it was mentioned, 20 residues were studied, and 56 mutants were generally constructed. Finally, three residues including Asn164, Pro190, and Val191 were selected since they have a close relationship with the catalytic cavity and the highest impact on improving cyanide hydratase docking. These three residues, Asn164, Pro190, and Val191, were mutated to Arg164, Gly190, and Cys191, respectively. A model of the mutant protein was predicted, and it was refined. mCHT contained 363 residues and its molecular weight was 41.3 KDa. The calculated isoelectric point (PI) of mCHT was 5.9, which indicated the acidic nature of the protein. Its high value of aliphatic index (81.13) is related to the probable stability of proteins in a wide range of temperatures. The instability index for mCHT is 39.38, indicating the probable stability of protein in a test tube [18]. The GRAVY indices were estimated to be −0.477. Analysis using Verify3D showed that 64.38% of amino acids produced a score that is above 0.2. Ramachandran plot analysis by PROCHECK showed that 98.9% of residues were within the most favored and allowed regions, while 1.1% were within the disallowed region (Figure 6A). The energy of structure analysis of mCHT using the ProSA web server showed that the z-score of the model is within the range of scores typically found for native proteins of similar size (Figure 6B). ERRAT quality score that takes non-bonded interactions into consideration was 75.2542, which is greater than 50 set as a threshold for high-quality structures [22] (Figure 6C). After its docking with CN^-^, there was a significant improvement in its docking (Table 4). The distance between the substrate and the catalytic triad in the mutant protein was analyzed, and it showed that the distance between a carbon atom of cyanide and sulfur atom of Cys191 in the active site was closer to the optimum distance than the amount of Cys165 in the mutant protein, and that Cys191 can play a key role as the protein active site (Table 5). Also, the surface charge density in the catalytic cavity indicates easier access for the carbon atom of the substrate and starting the reaction (Figure 7). The total charge of the mutant protein in pH 6.5 is −5.0004, while this amount for wild-type protein is −6.0004, and their isoelectric points are 5.77 and 5.9, respectively. Figure 6 shows the electrostatic potential molecular surface of these proteins and blue and red regions have positive and negative charge, respectively. The most important residues, pK (1/2), of wild-type and mutant proteins are indicated in Table 6.

### 2.4. Molecular Dynamics Simulation

**Figure 8:**
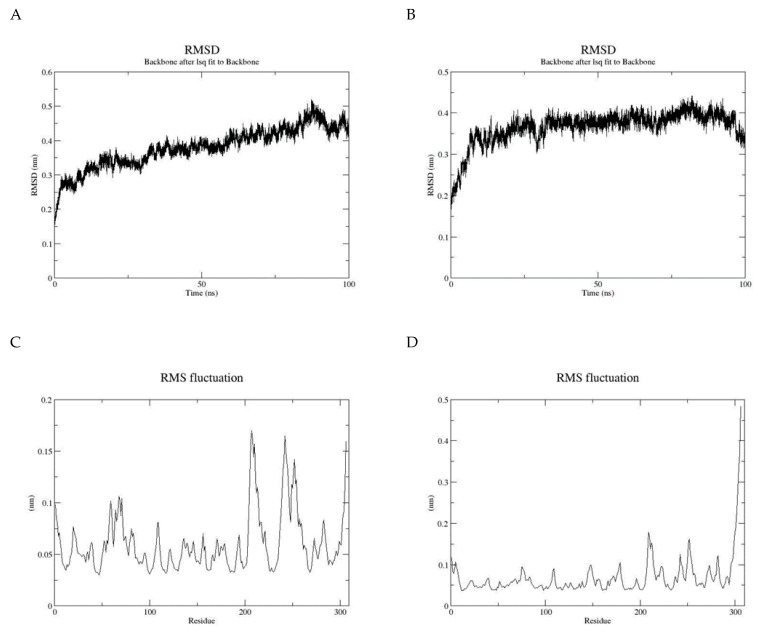
(**A**) Root-mean-square deviations (RMSDs) of Cα atoms for two models, CHT and (**B**) mutant of CHT (mCHT), (**C**) root-mean-square fluctuations (RMSFs) of side chains for CHT model, and (**D**) root-mean-square fluctuations (RMSFs) of side chains for mCHT model.

**Figure 9:**
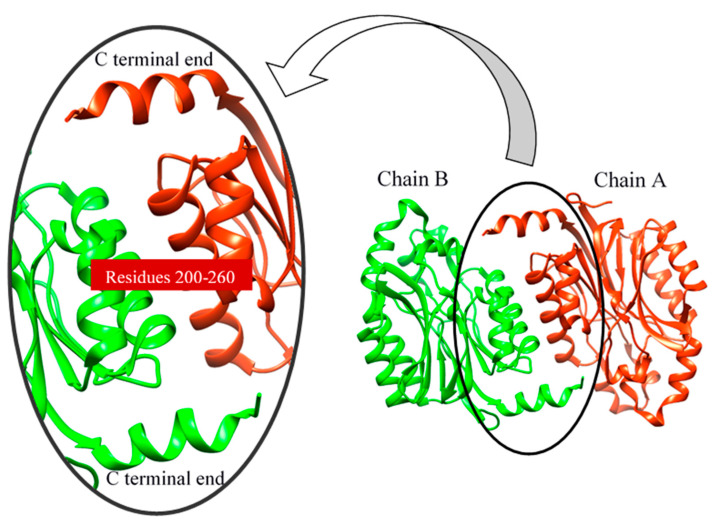
Interaction of two subunits (chain).

Molecular dynamics can be used to explore the conformational space and binding pose of the molecules in the active site. It was used to determine the quality and stability of the HCN binding to the active site of cyanide hydratase. The predicted docked structure of CHT and its mutant with CN^-^ was subjected to MD simulation for 100 ns to find out the level of conformational stability of the complex. Figure 8 shows the root-mean-square deviations (RMSDs) of Cα atoms for two models of CHT and mutant CHT (mCHT), and the RMSD is a quantitative measurement of the difference between two atomic structures. Backbone deviation of these proteins in bounded forms of the initial structure was plotted as a function of time. The bounded enzymes showed RMSD values in the range of 0.15 to 0.5 nm during simulation. As displayed in Figure 8a, the CHT-CN^-^ complex RMSD value is 0.4 at 50 ns, and then increased up to 0.5 at 90 ns. The RMSD value of mCHT-CN^-^ is 0.4 at 50 ns, after which it is stable. The average of deviations in the plateau region for mCHT was 4 Å and 40 ns, which are less for mCHT compared with CHT. As a result, it can be concluded that RMSD for CHT is higher than mCHT. This indicates that mCHT has higher level of conformational stability of the complex. We checked the flexibility of the backbone in the CHT complex compared to the mCHT complex through the root-mean-square fluctuation (RMSF) plot (Figure 8b). High RMSF value shows more flexibility, whereas the lower value indicates limited mobility during simulation. Figure 8 shows the root-mean-square fluctuations (RMSFs) of side chains for two models, CHT and mCHT (in the plateau region), and the RMSF is a measure of the deviation between the position of a particle and the reference position. It calculates according to GROMACS suite. Based on the obtained results, fluctuations follow a certain pattern, which can explain the observed trend in RMSD values (Figure 8). As indicated, residues 200–260 showed more flexibility in CHT and mCHT complexes (Figure 8c,d). The flexibility of movement in this region of proteins cannot interfere with substrate binding. In general, small degrees of fluctuation were observed for residues in the active site of these complexes, which indicated that the residues of active site in both complexes are well-stable when binding to the substrate. Significant fluctuations occur around Val, Pro, Asp, and Arg residues (residues 210, 245–280, 250, and 275). Because of the intermolecular interactions and conformational rearrangements between two subunits in the predicted model of homodimer proteins, high fluctuation of these side chains can occur (Figure 9).

## 3. Discussion

As a consequence of the cyanide usage in industrial activity and limitation in the number of cyanide-degrading enzymes, designing and introducing new enzymes with better functionality is critical. Despite numerous discovered and sequenced enzymes, there is a great demand for new enzymes with better performance in cyanide biodegradation. Predicted protein–ligand docking and interaction is essential for deep understanding of enzyme function analysis in an organism. In this study, we predicted a new enzyme from a cyanide hydratase of *Trichoderma harzianum* with three mutations. Structural bioinformatics approaches including multiple alignment, molecular docking, and molecular dynamics simulation were applied for an established procedure. The cyanide hydratase sequence of the indigenous *Trichoderma harzianum* has the highest identity with cyanide hydratase of *Trichoderma harzianum* (GeneBank accession no. KKO98780.1) in the sequence alignment. In this alignment, the conserved residues and residues that are involved in active sites were specified. In previous works, it has been shown that the Cys163 residue of cyanide hydratase and all the nitrilase-related sequences is conserved and is essential for enzyme activity [28,29]. In a study, the improvement of *Alcaligenes faecalis* Nitrilase was carried out by mutation of Gln196Ser and Ala284Ile, which leads to the highest activity and ability of tolerance to the substrate [30]. It has been revealed that cyanide hydratase of *Fusarium oxysporum* has the highest MolDock score by comparing different models of fungal species CHT, and the optimal distances of A and B were estimated to be 6.13 Å and 4.62 Å. Also, several studies showed that different strains of *Fusarium oxysporum,* such as *Fusarium oxysporum* CCF 1414, *Fusarium oxysporum* N-10, and *Fusarium oxysporum* CCF 483, can be useful in cyanide and nitrile degradation by cyanide hydratase and nitrilase [31,32]. Despite the current strategies for mutagenesis in nitrilase/cyanide hydratase and by introducing random mutations [33], a purposeful method was used in this work. The CHT model built based on 3wyu structure was confidently proceeded to the next steps. We docked CHT against CN^-^ retrieved from the ZINC database. As it was described, the model with the aim of cyanide hydratase was analyzed and the location of the mutations was determined for improving cyanide hydratase docking by three strategies, and these mutations are Asn164Arg, Pro190Gly, and Val191Cys. Also, in previous works, variants with multiple mutations were constructed, including Cys163Asn mutation and a deletion at the C terminus of the enzyme and/or the modification Ala165Arg. These constructs demonstrated increased amide formation capacity in comparison to the mutants carrying only single mutations [34]. Comparing docking results of mutant and wild-type proteins showed a significant improvement in the MolDock score, and the mutation in a direct neighborhood to the cysteine residue of the active site resulted in the MolDock score improvement of cyanide hydratase. Also, the A, B, and C distances in mutant protein were closer to the optimal distances than wild-type protein. As these distances become closer to the optimal distances, the docking results will become better. In analyzing charge distribution of the protein catalytic site, it was revealed that mutant protein has a more positive charge rather than wild-type protein, and this positive charge can be effective in attracting CN^−^ as a substrate, and it can facilitate the first step of cyanide degradation reaction. The purpose of Molecular Dynamic (MD) simulations was to investigate the stability of the CHT and mCHT structure and the state of fluctuation of different amino acids. The MD simulations analysis showed conformational stability of the CHT and mCHT complex, and the average of deviations in the plateau region for enzyme complexes was 4 Å in 50 ns. The highest level of flexibility of CHT and mCHT complexes was related to residues 200–260, which are in contact to other subunits. The results of molecular dynamic simulation (RMSD and RMSF) showed that the CHT and mCHT complexes have sufficient stability in docking analysis.

## 4. Materials and Methods

### 4.1. Cyanide Hydratase (cht) Identification, Multiple Sequence Alignment, and Sequence Analysis

All gene and protein sequences used in this study were obtained from the Protein Data Bank (PDB) and the National Center for Biotechnology Information (NCBI) (www.ncbi.nlm.nih.gov, accessed on 1 January 2020). Physical and chemical properties of CHT and mCHT, including molecular weight, isoelectric point, instability index, aliphatic index, and grand average hydropathy (GRAVY), were studied using ProtParam tools of Expasy (http://www.expasy.org, accessed on 1 January 2020) [35]. Secondary structure of CHT and mCHT protein was predicted and compared using the PSIPRED protein structure prediction server [36]. The examination of functional domains and motifs was done using Pfam [37], InterPro [38], and Conserved Domain Database (CDD) [39]. The cyanide hydratase proteins from *Aspergillus awamori* (Aacht) (GeneBank accession no.GCB25989.1), *Fusarium oxysporum* (Focht) (GeneBank accession no. RKL35637.1), *Fusarium solani* (Fscht) (GeneBank accession no. Q96UG7.1), *Micromonospora sp. L5* (Mcht) (GeneBank accession no. ADU09518.1), *Stemphylium lycopersici* (Slcht) (GeneBank accession no. RAR05160.1), *Trichoderma harzianum* (Hypocrea lixii) (Thhcht) (GeneBank accession no. KKO98780.1), and an indigenous *Trichoderma harzianum* isolated from Kerman (GeneBank accession no. MH629686) were collected and multiple sequence alignments were performed using ClustalW2 (ClustalW2 program: http://www.ebi.ac.uk/Tools/msa/clustalw2/, accessed on 1 January 2020) [40].

### 4.2. Homology Modeling and Docking Analysis

In order to find a known experimental structure as a template for homology modeling, the basic local alignment search tool for proteins (BLASTP) in NCBI was used and templates for structure modeling were selected according to the sequences’ identity, similarity, and coverage. As a result, the crystal structure of *Syechocystis* sp. Nitrilase (Nit6803) in Protein Data Bank (PDB; Acc No. 3wuy chain A) [23] was used as a template for homology modeling and models were constructed based on the crystal structures of nitrilase as a protein homodimer. Cyanide hydratase models were generated using the SWISS-MODEL workspace [41] and they were visualized using the well-known molecular modeling system, UCSF Chimera (http:// www.cgl.ucsf.edu/chimera/, accessed on 1 January 2020) [24]. Generated structures were improved by the subsequent refinement of the loop conformations by assessing the compatibility of an amino acid sequence to known PDB structures using the Galaxy web services [42]. The quality of the models was validated by PROCHECK [20], VERIFY3D (19), PROVE [43], and ERRAT [44], through the Structure Analysis and Verification Server (SAVES). The best-quality model was evaluated using ProTSAV (http://www.scfbio-iitd.res.in/software/proteomics/protsav.jsp, accessed on 1 January 2020), and the ProSA web service (https://prosa.services.came.sbg.ac.at/prosa.php, accessed on 1 January 2020) [21] was selected for further calculations, molecular modeling, and docking studies using the Molegro Virtual Docker (MVD) version 4.0.2 [25]. The 3D structure of the enzyme substrate was downloaded from the ZINC database [45] and energy minimization of the substrate was done using MVD, and the optimized structure was saved for further analysis through molecular docking [46]. Charge distribution over the entire molecule surface was calculated using PyMol and the protonation state of key residues was investigated using the H++ web server [47,48].

### 4.3. Constructing a Mutant of Cyanide Hydratase, Modeling, and Comparing with Wild-Type Enzyme

New construction of cyanide hydratases was designed by computational design methods that focus on mutants for constructing a new model of cyanide hydratase with better docking for cyanide. These mutations were created by swiss Pdbviewer software [49]. Twenty residues were studied, which were in contact with the active site of cyanide hydratase, and three designed mutations were selected and combined, based on three criteria: reduction of the steric hindrance, enhancing nucleophilicity and nucleophilic attack to the carbon atom of cyanides, and increasing positive charge in the active site. The homology modeling for the newly designed mutant was carried out by the SWISS-MODEL workspace [41] and was evaluated using the ProSA web service [21]. The docking analysis was studied for the mutant using the Molegro Virtual Docker software [25] and was compared with the wild-type.

### 4.4. Molecular Dynamics Simulation

Molecular dynamics (MD) simulation was applied to examine the stability of the protein–ligand complex in GROMACS suite [50]. Both of the models, CHT and mCHT, were simulated in the presence or absence of the ligand, in two separate runs. Afterward, GROMOSE 54A7 forcefield [51] was used to create proper topologies. The models were placed at the center of a dodecahedral box and solvated with the TIP3P water model, while Cl^−^ or Na^+^ ions were used for the neutralization of each system. The energy of the system was minimized with the steepest descent method to eliminate possible clashes and bad contacts. Subsequently, the equilibrations of systems were done under NVT up to 100 ps at 300 K with restraint forces of 1000 kJ/mol, followed by 100 ps under NPT at the pressure of 1 bar, and with restraint forces of 1000 kJ/mol. The electrostatic interactions were calculated using the Particle Mesh Ewald (PME) method [52]. Finally, a 100 ns MD run with no restraint was performed for evaluating the systems’ stability. The root-mean-square fluctuations (RMSFs) and the root-mean-square deviations (RMSDs) were analyzed for the wild-type enzyme and its mutant in the MD simulation [53].

## 5. Conclusions

The present study aimed to analyze the structure of CHT and design mutations for this enzyme for performance improvement. Toward performance analysis of CHT and comparing with mCHT, docking analysis with cyanide as a substrate was done, and this analysis showed a great improvement. The MD simulation showed that cyanide as a substrate can bind stably to CHT and mCHT, interacting with the conserved residues in active sites. The data showed that accessibility to the best mutations for improved enzyme performance can be useful in bioremediation and they can play a bridge role in connecting genomic sequences, structural bioinformatics, and systems biology, with the purpose of introducing new and efficient enzymes for cyanide bioremediation. For further analysis on the enzyme–ligand interaction, it is recommended that future work should be focused on employing the quantum mechanical and molecular mechanical (QM/MM) calculations to study the catalytic reaction of CHT and mCHT. Furthermore, to study and evaluate the transition state stabilization in the enzyme active site, the QM/MM calculations can be used, and this analysis can assist future research in making a better comparison between the wild-type enzyme and its mutant.

## Figures and Tables

**Table 1:** Cyanide hydratase of different fungi and their MolDock score.

Fungi Containing Cyanide Hydratase	MolDock Score
*Aspergillus awamori*	−23.6224
*Fusarium oxysporum*	−25.2489
*Fusarium solani*	−22.9649
*Micromonospora sp. L5*	−21.0853
*Stemphylium lycopersici*	−18.8413
*Trichoderma harzianum*	−24.44
Indigenous *Trichoderma harzianum*	−18.1752

**Table 2:** The residues that have a close relationship with catalytic cavity using Molegro Virtual Docker software.

Type of Residues	Number of Residues	Type of Residues	Number of Residues
Tyr	54	Val	191
Tyr	56	Tyr	192
Lys	132	Pro	193
Thr	134	Pro	194
Asn	164	Ala	195
Cys	165	Tyr	196
Trp	166	Gln	199
Glu	167	Tyr	200
Asn	168	Pro	201
Pro	190	Tyr	204

**Table 3:** The A, B, and C distances in different fungal strains.

Fungi Containing Cyanide Hydratase	A^1^	B^2^	C^3^
*Aspergillus awamori*	7.70 Å	1.39 Å	6.58 Å
*Fusarium oxysporum*	6.13 Å	4.62 Å	3.98 Å
*Fusarium solani*	8.34 Å	3.76 Å	9.57 Å
*Micromonospora sp. L5*	9.23 Å	4.39 Å	12.76 Å
*Stemphylium lycopersici*	3.77 Å	3.60 Å	3.09 Å
*Trichoderma harzianum*	6.12 Å	4.05 Å	5.36 Å
*Indigenous Trichoderma harzianum*	7.34 Å	4.61 Å	6.57 Å

A^1^. The distance between the nitrogen atom of cyanide and the hydrogen atom of Lys in the active site. B^2^. The distance between the carbon atom of cyanide and sulfur atom of Cys in the active site. C^3^. The distance between the oxygen atom of Glu and hydrogen atom of Cys in the active site.

**Table 4:** MolDock score comparison in wild-type and mutant protein of cyanide hydratase.

Type of Protein	Wild Type Protein	Mutant Protein
Moldock Score	−18.1752	−23.8575

**Table 5:** The distance between substrate and catalytic triad in the wild-type and mutant protein.

Cyanide Hydratase of Indigenous *Trichoderma harzianum*	A^4^	B^5^	C^6^
Wild-Type	7.34 Å	4.61 Å	6.57 Å
Mutant	7.22 Å	5.00 Å of Cys165 and 4.52 Å of Cys191	6.57 Å

A^4^. The distance between Nitrogen atom of cyanide and Hydrogen atom of Lys in active site. B^5^. The distance between carbon atom of cyanide and sulfur atom of Cys in active site. C^6^. The distance between Oxygen atom of Glu and Hydrogen atom of Cys in active site.

**Table 6:** The most important residues, pKa, of wild-type and mutant proteins.

Mutant Protein	Native Protein
Glu48: <0.0	Glu48: 3.441
Lys130: <0.0	Lys130: 0.483
Cys165: >12.0	Cys165: >12.0

## Data Availability

Data is contained within the article.

## Sample Availability

Not aplicitable.
